# Ultrasound treatment enhanced the functional properties of phycocyanin with phlorotannin from *Ascophyllum nodosum*

**DOI:** 10.3389/fnut.2023.1181262

**Published:** 2023-04-06

**Authors:** Ying Bai, Xueting Li, Yuqianqian Xie, Yingzhen Wang, Xiuping Dong, Hang Qi

**Affiliations:** ^1^School of Food Science and Technology, National Engineering Research Center of Seafood, Liaoning Provincial Aquatic Products Deep Processing Technology Research Center, Dalian Polytechnic University, Dalian, China; ^2^Haide College, Ocean University of China, Qingdao, China

**Keywords:** ultrasound, phycocyanin, phlorotannin, *Ascophyllum nodosum*, interaction, modification

## Abstract

**Introduction:**

Phycocyanin offers advantageous biological effects, including immune-regulatory, anticancer, antioxidant, and anti-inflammation capabilities. While PC, as a natural pigment molecule, is different from synthetic pigment, it can be easily degradable under high temperature and light conditions.

**Methods:**

In this work, the impact of ultrasound treatment on the complex of PC and phlorotannin structural and functional characteristics was carefully investigated. The interaction between PC and phlorotannin after ultrasound treatment was studied by UV–Vis, fluorescence spectroscopy, circular dichroism (CD) spectroscopy, fourier transform infrared (FTIR) spectroscopy. Additionally, the antioxidant potential and *in vitro* digestibility of the complexes were assessed.

**Results:**

The result was manifested as the UV–Vis spectrum reduction effect, fluorescence quenching effect and weak conformational change of the CD spectrum of PC. PC was identified as amorphous based on the X-ray diffraction (XRD) data and that phlorotannin was embedded into the PC matrix. The differential scanning calorimetry (DSC) results showed that ultrasound treatment and the addition of phlorotannin could improve the denaturation peak temperatures (Td) of PC to 78.7°C. *In vitro* digestion and free radical scavenging experiments showed that appropriate ultrasound treatment and the addition of phlorotannin were more resistant to simulated gastrointestinal conditions and could improve DPPH and ABTS+ free radical scavenging performance.

**Discussion:**

Ultrasound treatment and the addition of phlorotannin changed the structural and functional properties of PC. These results demonstrated the feasibility of ultrasound-assisted phlorotannin from *A. nodosum* in improving the functional properties of PC and provided a possibility for the application of PC-polyphenol complexes as functional food ingredients or as bioactive materials.

## 1. Introduction

In recent years, the development and efficient utilization of functional protein has become a research hotspot for economic, ecological and health reasons (1). There has been a resurgence of interest in the food-approved blue phycobiliprotein phycocyanin (PC), which accumulated up to 47.7% under controlled culture conditions (2, 3), and it is a supramolecular protein-chromophore complex made up of polypeptides with α- and β-subunit. Under various environmental conditions, the essential energy transfer units are formed when the (αβ)-monomers, which function as the building blocks, oligomerize into (αβ)_3_-trimers or (αβ)_6_-hexamers (4, 5). It is also a water-soluble, blue pigment (6). In addition to being used as a pigment in food product formulations (one of the limited choices for natural light blue-colored dyes), PC also offers advantageous biological effects, including immune-regulatory, anticancer, antioxidant, and anti-inflammation capabilities (7, 8). However, PC, as a natural pigment molecule, is different from synthetic pigment. It can be easily degradable under high temperature and light conditions, which might influence the appearance and overall quality of the product.

Polyphenols might interact with proteins by either noncovalent interactions or covalent bonding (9). Numerous studies have been done on the noncovalent bonding between proteins and polyphenols. For instance, polyphenol hydroxyl groups engage in hydrogen bonding interactions with polar polypeptide groups, whereas polyphenols with hydrophobic substituent groups bind proteins through hydrophobic interactions (10, 11). Protein and polyphenol interactions may alter the taste, color, and nutritional value of food as well as the way it is absorbed and digested (5). Previous researchers have reported that other polyphenols, including rosmarinic acid, chlorogenic acid, gallic acid, and epigallocatechin gallate, can interact with protein and modulate its molecular, physicochemical, and functional properties (12). Phlorotannin extracted from brown algae is a phenolic compound and a derivative of phloroglucinol. However, it is worth studying whether phlorotannin could be used to improve the stability of PC. To explore the combination of phlorotannin and PC and to carry out the functional characteristics, so as to expand the application of PC as a natural blue pigment in the food industry (1).

As a safe, nontoxic and environmentally friendly technology, ultrasound has been used in the food industry (13). Due to the thermal and cavitation effects, low frequency ultrasound generates substantial shear and mechanical forces (14). These forces may alter the protein’s molecular structure, hydrogen bonding, and hydrophobic interactions, which would alter the protein’s physicochemical and functional characteristics (15) and enhance its capacity to bind to polyphenols.

In this study, with the aid of ultrasound treatment, the interaction between PC and phlorotannin from *A. nodosum* was investigated. Fourier transform infrared (FTIR) spectroscopy, differential scanning calorimetry (DSC), and fluorescence spectroscopy were used to thoroughly examine the structural alterations of the complex and the impact of the ultrasound treatment on the complex’s formation mechanism. Additionally, we assessed the complexes’ antioxidant potential and *in vitro* digestibility, which offered a fresh theoretical framework and practical instructions for the exploration of protein-polyphenol complexes.

## 2. Materials and methods

### 2.1. Materials and reagents

PC was purchased from Zhejiang Binmei Biotechnology Co., Ltd (Zhejiang Province, China). Phlorotannin was extracted in accordance with the procedure described by Shen et al. (16). 2,2′-Azino-bis (3-ethylbenzothiazoline-6-sulfonic acid) (ABTS) was obtained from Sigma Chemical Co. (St. Louis, MO, United States). Fetal bovine serum (FBS) was obtained from Shenggong Bioengineering Co., Ltd (Shanghai, China). All other chemicals were of analytical grade.

### 2.2. Ultrasound-assisted preparation of PC-phlorotannin complexes (PP)

The powdered PC was dissolved in deionized water to achieve a 0.2% (w/v) PC solution. Phlorotannin was dissolved in 0.02 M phosphate buffer solution (pH 7.4) to obtain 0.05–0.2% (w/v) phlorotannin. To obtain the PP, the PC solution was completely mixed with an equal amount of phlorotannin solution; subsequently, the mixture was subjected to ultrasound treatment (Scientz Biotechnology Co., Ltd., Ningbo, China) at an amplitude of 40%, frequency of 20 kHz and power of 250 W for 2 min. The mixture was incubated for 2 hours at 25°C in the dark to initiate complexation (UPP) (17, 18).

### 2.3. Characterization

#### 2.3.1. Determination of UV–vis spectra

The UV–vis absorption spectra of PP and UPP with different mass ratios (1:0, 1:0.25, 1:0.5, 1:1, PC/phlorotannin) were analyzed in the range of 200–800 nm with a spectrophotometer (UV-2600 UV–VIS, Shimadzu, Tokyo, Japan). The protein concentration in all samples was 0.2 mg/mL.

#### 2.3.2. Determination of the intrinsic emission fluorescence spectrum

The fluorescence spectrum was obtained based on the slightly adjusted procedure from Zhang et al. (19). The protein concentration of the samples was 0.2 mg/mL. Using a fluorescence spectrophotometer (Hitachi F-2700, Hitachi, Tokyo, Japan), the intrinsic fluorescence was examined at a constant excitation wavelength of 295 nm with a slit width of 10 nm and an emission wavelength ranging from 310 to 400 nm.

#### 2.3.3. Sodium dodecyl sulfate-polyacrylamide gel electrophoresis (SDS-PAGE)

SDS-PAGE analysis of the samples was performed under reducing conditions using the method of Nooshkam & Varidi with slight modifications (20). PC, phlorotannin, PP, and UPP were separated by SDS-PAGE using a 5% condensation gel and a 12% separation gel. The voltage was set at 80 and 120 volts, respectively, for sample condensation and sample separation. At the end of the electrophoresis, the electrophoretic film was stained, decolorized, and then imaged with a ChemiDoc Touch Imaging System (ChemiDoc Touch, Bio-Rad, California, United States).

#### 2.3.4. Determination of circular dichroism (CD)

A slightly modified procedure of Bai et al. was used for the CD experiments (21). The mixtures were diluted to 0.05 mg/mL protein solutions before being placed in a 10 mm quartz sample cell. Following that, the samples were scanned within 190 and 260 nm at a speed of 100 nm/min with a bandwidth of 1 nm. For background rectification, distilled water was scanned as a control.

#### 2.3.5. Determination of fourier transform infrared (FTIR) spectroscopy

Using a Fourier Transform Infrared Spectrometer (FTIR) (PerkinElmer, Norwalk, United States), the FTIR spectra of the samples were measured. Freeze-dried samples were combined with potassium bromide (KBr) and the results were measured with a 4 cm^−1^ resolution between 4,000 and 400 cm^−1^ (22).

#### 2.3.6. Differential scanning calorimetry (DSC) analysis

The thermal behavior of the samples (PC, PP, and UPP) was investigated by DSC (DSC250, TA Instrument, New Castle, DE, United States) under a nitrogen atmosphere following the method reported by Hu (23). The samples (6.0–10.0 mg) were put in an aluminum pot and sealed with an aluminum lid. The heating temperature was 20–100°C, the heating rate was 3°C/min, and the dry nitrogen purging rate was 50 mL/min. Running an empty aluminum pan was used as a baseline. The denaturing peak temperature (Td) of each thermal curve was calculated by using general analysis software.

#### 2.3.7. X-ray diffraction (XRD)

The XRD patterns of PC, phlorotannin, PP and UPP were recorded using a XRD 6100 (Shimadzu, Japan). Copper Kα was used in this process, while the current and voltage were set at 30 kV and 20 mA, respectively. The scanning speed was maintained at 8°/min and the range of the diffraction angle was 10–80°.

### 2.4. Scanning electron microscopy

The freeze-dried samples were mounted on circular aluminum stubs and sputter-coated with a gold layer. The surface morphology of PC, phlorotannin, PP and UPP was studied using a scanning electron microscope (SU8010, Hitachi, Tokyo, Japan).

### 2.5. *In vitro* digestion

PC, PP and UPP were diluted properly with ultrapure water to reach a protein concentration of 20 mg/mL. After being incubated for 10 min at 37°C, 1 mL of a freshly prepared dispersion was sequentially mixed with 5 mL of the simulated gastric fluid (SGF, pH 2 with 1 mg/mL pepsin), shaken continuously at 100 rpm and 37°C for 2 h, mixed with 5 mL of the simulated intestinal fluid (SIF, pH 7.5 with 10 mg/mL pancreatin), and shaken continuously at 100 rpm and 37°C for an additional 4 h (24).

Furthermore, SDS-PAGE was carried out to analyze the pepsin-pancreatin digests according to the method described in Section 2.3.3.

### 2.6. Free radical scavenging activity

The DPPH radical scavenging activity of PC, phlorotannin, PP, and UPP was measured by electron spin resonance spectroscopy (ESR). Briefly, DPPH was dissolved in 95% ethanol to obtain a 200 μmol/L solution. Next, PBS, DPPH, and sample solution were added to the 2-mL tubes and reacted in the dark for 30 min. A certain amount of supernatant was sucked into the capillary, put into the resonance chamber, and measured. Distilled water was used as a blank control, and the relative intensity of the signal was represented by the third peak height of the spectral signal. ESR measurements were carried out using a Bruker EMX A200 (Bruker, A200, Germany). The parameters employed were as follows: center field, 3,480 G; sweep width, 50 G; conversion time, 0.32 ms; time constant, 0.01 ms; modulation amplitude, 1 G. This assay was carried out under dark conditions. DPPH radical-scavenging activity was calculated according to the following formula (1):

(1) $$\mathrm{Free radical scavenging activity}\;\left(\%\right)=\left(1-\frac{\mathrm{As}}{\mathrm{Ac}}\right)\times 100$$

where As and Ac stand for, respectively, the sample’s and the control’s absorbance.

With a few minor adjustments, the method described by Liu et al. (25) was used to assess the radical scavenging activity of ABTS^+^. Potassium persulfate (2.45 mM) was mixed with an equal volume of ABTS (7 mM) and allowed to stand for 16 h in the dark. The combined solution was turned into ABTS^+^ radical solution by diluting it with 0.2 M phosphate buffer solution (pH 7.4). 20 μL of samples (0.1–1 mg/mL) were mixed with 1,000 μL of ABTS^+^ radical solution and reacted for 6 min under dark conditions. Then, the absorbance of the mixed sample was measured by an Infinite 200 multimode microplate reader (Tecan 200, Hombrechtikon, Switzerland) at 734 nm. The ABTS^+^ radical scavenging activity of the sample was estimated using Eq. (2).

(2) $${\mathrm{ABTS}}^{+}\;\mathrm{radical scavenging activity}\left(\%\right)=\left(1-\frac{A_{1}}{A_{0}}\right)*100$$

where A_0_ denotes the sample solution’s absorbance and A_1_ denotes water’s absorbance.

### 2.7. Cell culture and treatment

Retinal Müller cells (RMCs) were used for experiments (24). Cells were seeded in 96-well plates for 24 h, and then three processes were performed on them: Procedure 1, fresh medium containing PC, phlorotannin, and UPP; Procedure 2, H_2_O_2_ and fresh medium containing PC and UPP; and Procedure 3, UVB and fresh medium containing PC and UPP for another 24 h. An Infinite 200 multimode microplate reader (Tecan 200, Hombrechtikon, Switzerland) was used to test the MTT assay at 490 nm to assess the viability of the cells (26) 2.8 Lipid Peroxidation.

Malondialdehyde (MDA) levels were used as a basis for measuring the lipid peroxidation activity. The RMCs in the 6-well plates incubated with procedure 3, UVB and fresh medium containing PC and UPP were obtained to measure the MDA levels. The cellular activity levels of MDA were determined using commercial kits (MDA, A003-1, Jiancheng Bioengineering Institute, Nanjing, China).

### 2.8. Statistical analysis

At least three times of each experiment were performed before the variance was examined. The mean ± standard deviation were used to describe the results. Using SPSS 16.0 (SPSS Inc., 2001, Chicago, IL, United States), all data were evaluated using one-way analysis of variance (ANOVA), with *p* < 0.05 being considered significant.

## 3. Results

### 3.1. Characterization

#### 3.1.1. UV/Vis absorption

**Figure 1:**
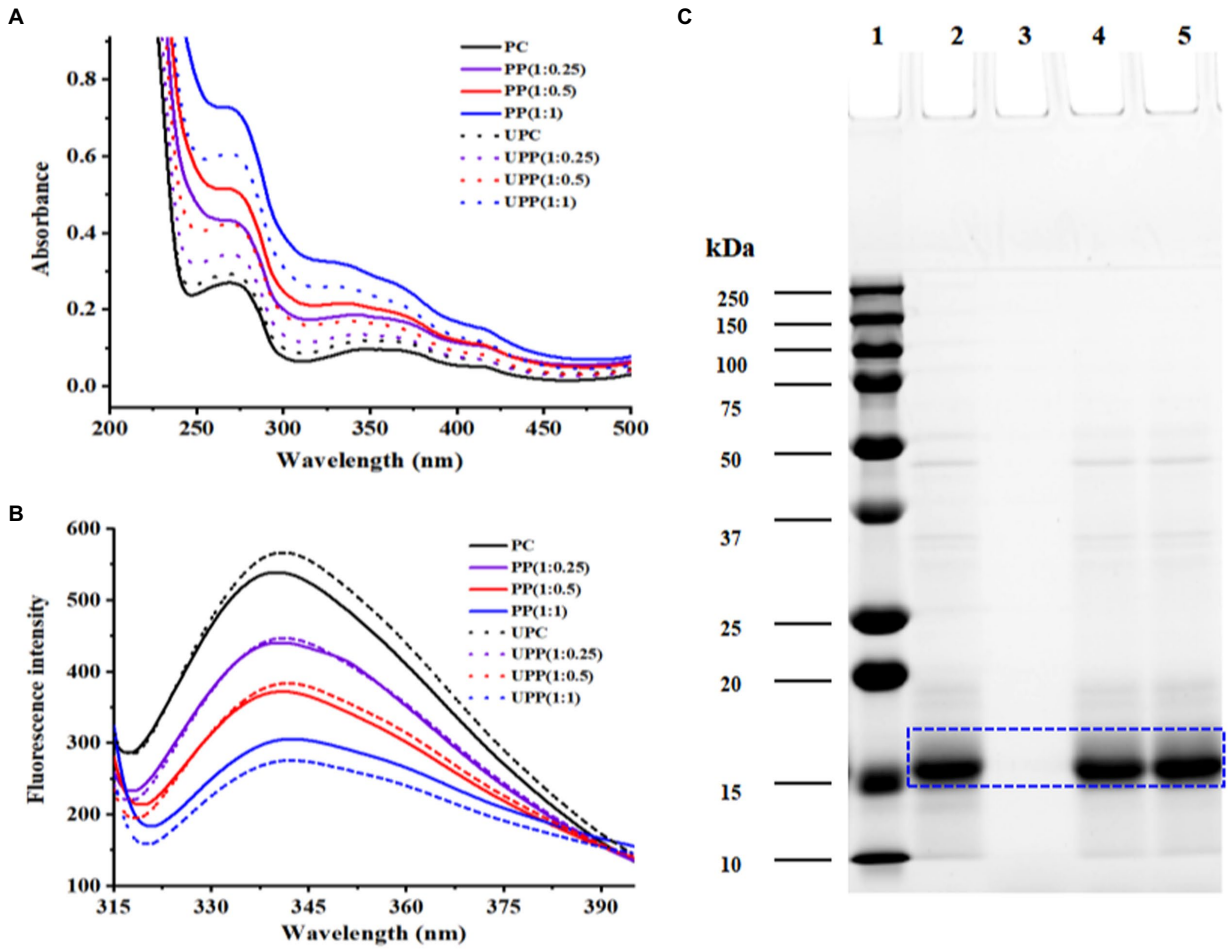
**(A)** UV–visible absorption spectra and **(B)** Fluorescence intensity of PC, PP (1:0.25), PP (1:0.5), PP (1:1), UPC, UPP (1:0.25), UPP (1:0.5), UPP (1:1), and **(C)** SDS-PAGE of 1-marker, 2-PC, 3-phlorotannin, 4-PP (1:1), 5-UPP (1:1).

The results from Figure 1A showed the UV/Vis spectra of PP and UPP (with different mass ratios of PC/phlorotannin = 1:0, 1:0.25, 1:0.5, and 1:1) from 200 to 800 nm. PC contained three typical absorption peaks at approximately 280 nm, which were ascribed to aromatic amino acid residues (27), 360 nm, and 620 nm. A minor redshift caused by phlorotannin and ultrasound treatment revealed the change of the aromatic amino acid residues in proteins to a more hydrophobic. A minor redshift caused by phlorotannin and ultrasonic support revealed the change of the aromatic amino acid residues in proteins to a more hydrophobic surrounding (28). When the mass ratio of PC/phlorotannin was 1:1, the absorption peak at approximately 360 nm disappeared. It could be concluded that the conformation of PC could be affected by binding with phlorotannin and ultrasound assistance (28).

#### 3.1.2. Fluorescence spectroscopy

The tertiary structure of proteins can be modified using the intrinsic fluorescence as a valuable indicator. It is based on how tryptophan residues alter in their surroundings (29). Tyrosine residues have low emission intensities, hence tryptophan residues are crucial in determining the fluorescence peak wavelength, which ranges from 320 to 350 nm. As shown in Figure 1B, ultrasound treatment increased the fluorescence intensity of PC. The increased fluorescence intensity indicated that the protein structure unfolded, the originally buried aromatic amino acid residues were exposed on the protein surface, and the polarity of the microenvironment increased (30). With increasing phlorotannin concentration, the fluorescence intensity of PC gradually decreased, and the change in the peak was small. Due to the interaction between PC and phlorotannin, which caused fluorescence quenching, the intrinsic fluorescence intensity decreased, suggesting that Trp may have been involved. These results were consistent with earlier research that claimed the presence of phlorotannin exposed Trp residues and caused proteins to unfold, which decreased the fluorescence intensity (31, 32).

#### 3.1.3. SDS-page

The electrophoretic protein profiles of PC, PP, and UPP were shown in Figure 1C. Major protein bands were identified at approximately 17, 34 and 48 kDa, of which 17 kDa was the most abundant in PC, PP, and UPP. For the PP complex, the intensity of the 34 and 48 kDa bands decreased, while the intensity of the 17 kDa bands increased. After ultrasound treatment, the intensity of the 34 and 48 kDa bands also decreased, but the intensity of the 17 kDa bands increased significantly. Phlorotannin and ultrasound treatment were found to increase the protein’s molecular weight by about 17 kDa, showing that the treatment caused big molecular weight proteins to degrade into small molecular structures. This event is in line with the findings of earlier research (13), which indicated that the cavitation impact changed the protein’s molecular structure. Chen et al. found a decrease in the intensity of whey protein isolate bands when investigating the formation of conjugates between whey protein isolate with gum acacia, and near the top of the gel, a new band with higher molecular weight appeared. The intensity of the whey protein isolate bands steadily reduced with increasing ultrasound time, and the bands near the top of the gel became more visible (33).

#### 3.1.4. Secondary structure analysis

**Figure 2:**
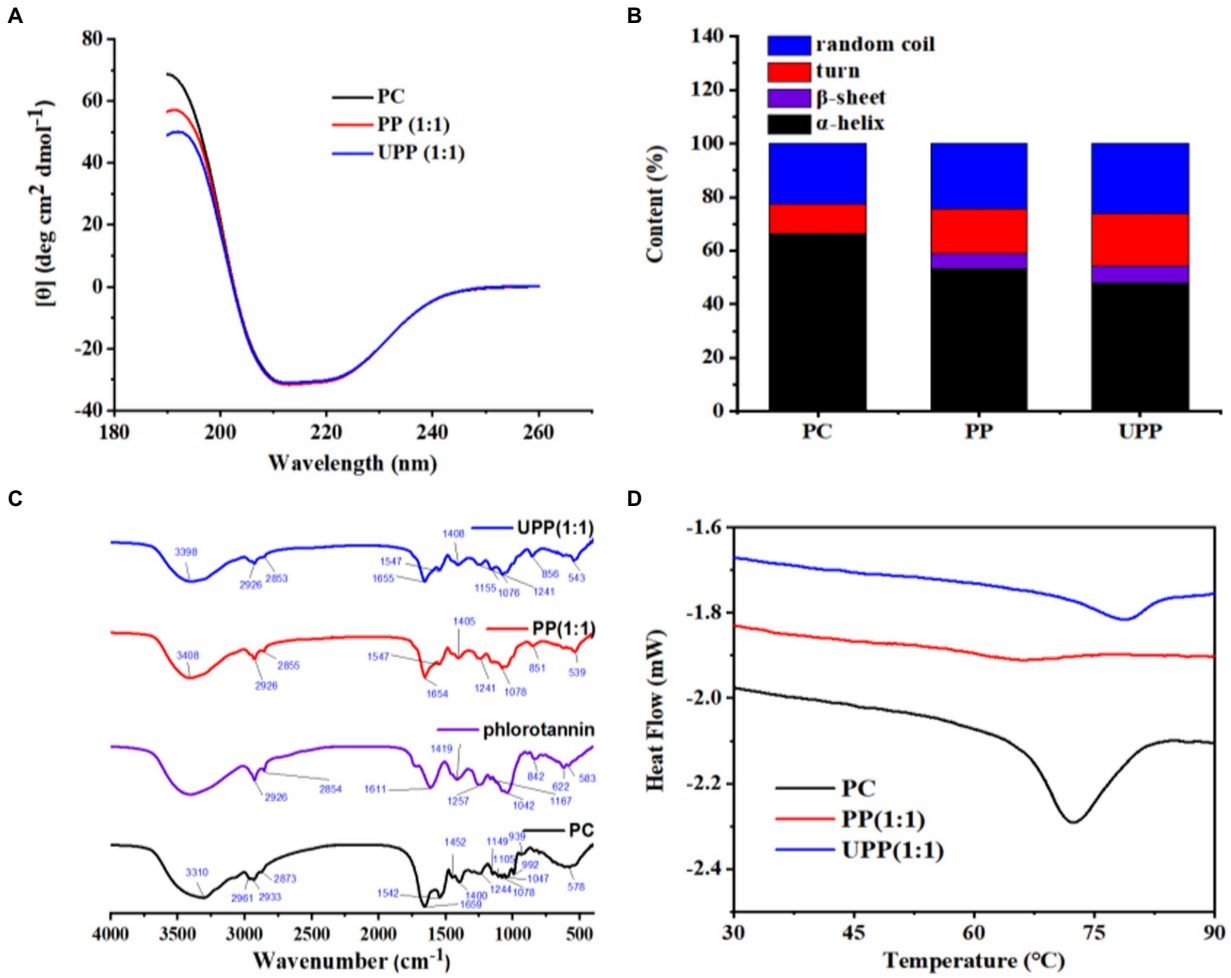
**(A)** CD spectra of PC, PP (1:1), UPP (1:1), **(B)** Protein secondary structure content, **(C)** FTIR spectra of PC, phlorotannin, PP (1:1), UPP (1:1), and **(D)** DSC thermogram of PC, PP (1:1), UPP (1:1).

CD can characterize ligand-induced protein conformational changes. As shown in Figure 2A, all samples have a peak at approximately 192 nm and a broad peak at approximately 211 nm. The protein secondary structure content is shown in Figure 2B. The PP complex’s α-helix content was lower than that of PC, while its β-sheet, β-turn and random coil were higher. Following ultrasound treatment, the secondary structure of UPP dramatically differed from the untreated PP complex; the amount of α-helix dropped, while the amounts of β-sheet, β-turn and random coil structures rose. This demonstrated that the UPP structure was more slacked and stretched, exposing the hydrophobic and polar groups within proteins to the surface. The molecular structure of the protein molecule loosened as a result of the hydrogen bond that stabilized the protein structure being broken, the protein molecule’s having less ordered structure, and the PC unfolding. The hydrophobic surface of PC was exposed to phlorotannin, which disrupted the hydrogen bond network there and reduced the amount of α-helix (32). Different studies have reported that the changes in the secondary structures after the interaction between proteins and polyphenols and ultrasound treatment were different (34–36). Therefore, the secondary structure change was not completely regular. Phlorotannin and ultrasound treatment both had an impact on PC’s secondary structure, causing the secondary structure of the complex to change irregularly.

#### 3.1.5. FTIR spectroscopy analysis

FTIR spectroscopy could provide information about chemical structure. In this study, the effects of ultrasound treatment and phlorotannin binding on the structural changes of PC were studied with PC and phlorotannin as controls. As shown in Figure 2C, PC had a characteristic peak at 3310 cm^−1^, while the peak in PP shifted to 3,408 cm^−1^ and the peak in UPP shifted to 3,398 cm^−1^, indicating that PC and phlorotannin interact through hydrogen bonds. By disrupting the intermolecular hydrogen bond and boosting protein flexibility, the cavitation impact of ultrasound treatment altered the protein secondary structure (37). These phenomena indicated that hydrogen bonding interactions existed in the complex. Most frequently, changes in protein secondary structure are reflected by the amide I band (1600–1700 cm^−1^) (38). No new distinctive peak occurred throughout the complexation process, implying that the formation of the complex was just the transfer of chemical bonds. The C-O stretching and N-H bending of amide bonds are mostly represented by the amide I (1700–1,600 cm^−1^) and amide II (1550–1,450 cm^−1^) bands, respectively (39). The amide I and II band peaks of PC and PP were 1,659 and 1,542 cm^−1^, and 1,654 and 1,547 cm^−1^, respectively. The amide I and II band peaks for UPP, however, migrated to 1,655 and 1,547 cm^−1^, respectively. The reason might be that electrostatic interactions led to changes in the FTIR bands of the complex (40). In addition, peaks in the range 2,890 to 2,980 cm^−1^ indicated the presence of C-H vibrations, indicating the presence of hydrophobic interactions (41).

#### 3.1.6. Thermal property

DSC is an efficient thermal analytic tool for examining changes in the thermal stability of PC and phlorotannin binding (42). Denaturation peak temperatures (Td) commonly indicate the thermal stability of a protein. As shown in Figure 2D, PC showed a Td at 72.35°C. After physical mixing with phlorotannin, PP exhibited a Td at 66.16°C, which was lower than that of PC, showing that the stability of the PC tertiary conformation decreased after covalently interacting with phlorotannin. Compared with PC and PP, UPP presented a higher Td at 78.7°C. The increased thermal stability of UPP might be due to the formation of hydrogen bonds,electrostatic and hydrophobic interactions, which was verified in the FTIR results. Following the conjugation of chlorogenic acid-lactoferrin-dextran, Liu et al. discovered that the thermal denaturation temperature of lactoferrin increased [42]. Various methodologies and ligands for polyphenols or polysaccharides could cause the changed protein to exhibit unique thermal behavior (43). It was noteworthy that ultrasound treatment and phlorotannin could be a method for the improvement in the thermal properties of PC.

#### 3.1.7. XRD analysis

**Figure 3:**
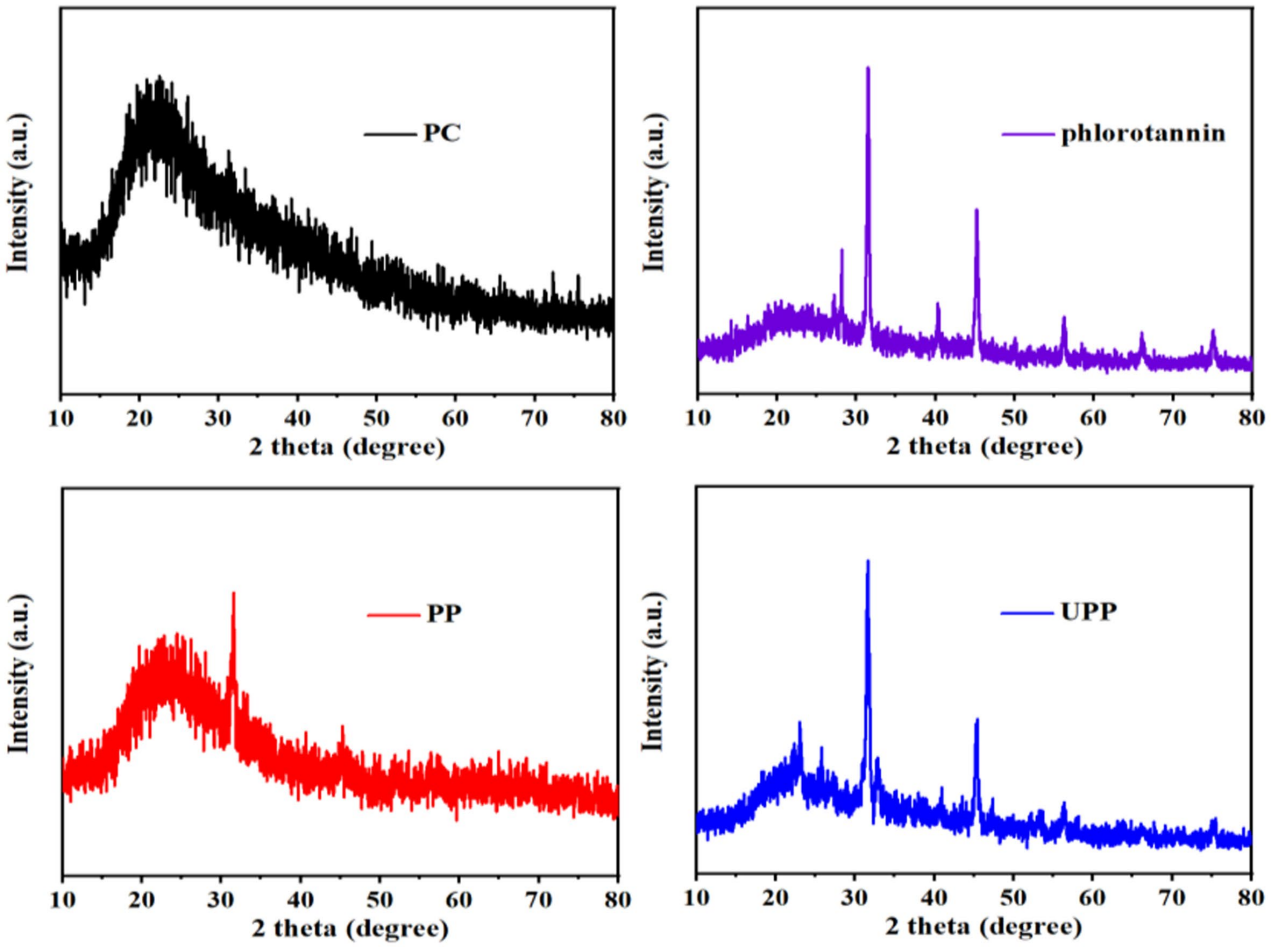
XRD patterns of PC, phlorotannin, PP (1:1), UPP (1:1).

The structural phases of different samples were analyzed by XRD analysis. The XRD spectras of PC, phlorotannin, PP, and UPP were displayed in Figure 3. There were no characteristic peaks in the XRD spectra for PC, indicating that PC was in an amorphous state (5). Phlorotannin possessed some spikes at 2θ = 27.12 °, 28.20 °, 31.56 °, 40.46 °, 45.26 °, 56.20 °, 66.08 ° and 75.08 °, corresponding to its high degree of crystallinity. When PC and phlorotannin formed a complex, the diffraction peaks of phlorotannin were partially covered, exhibiting only one distinct spike. This result indicated that phlorotannin was partially incorporated into the matrix created by PC and formed a partially crystalline structure. After ultrasound-assisted binding of phlorotannin to PC to form UPP, there were two distinct spikes. These results indicated that PP was broken by ultrasound and that the phlorotannin crystals embedded in PC were partially exposed. Sun et al. showed that the higher the diffraction peak intensity, the more amorphous structures were formed (32). Zhao et al. showed that ultrasound treatment had almost no effect on the crystalline region, but it could destroy the rigid structure and form more amorphous structure (34, 44). The increase in UPP diffraction peaks could be explained by the formation of an amorphous structure caused by ultrasound.

### 3.2. SEM analysis

**Figure 4:**
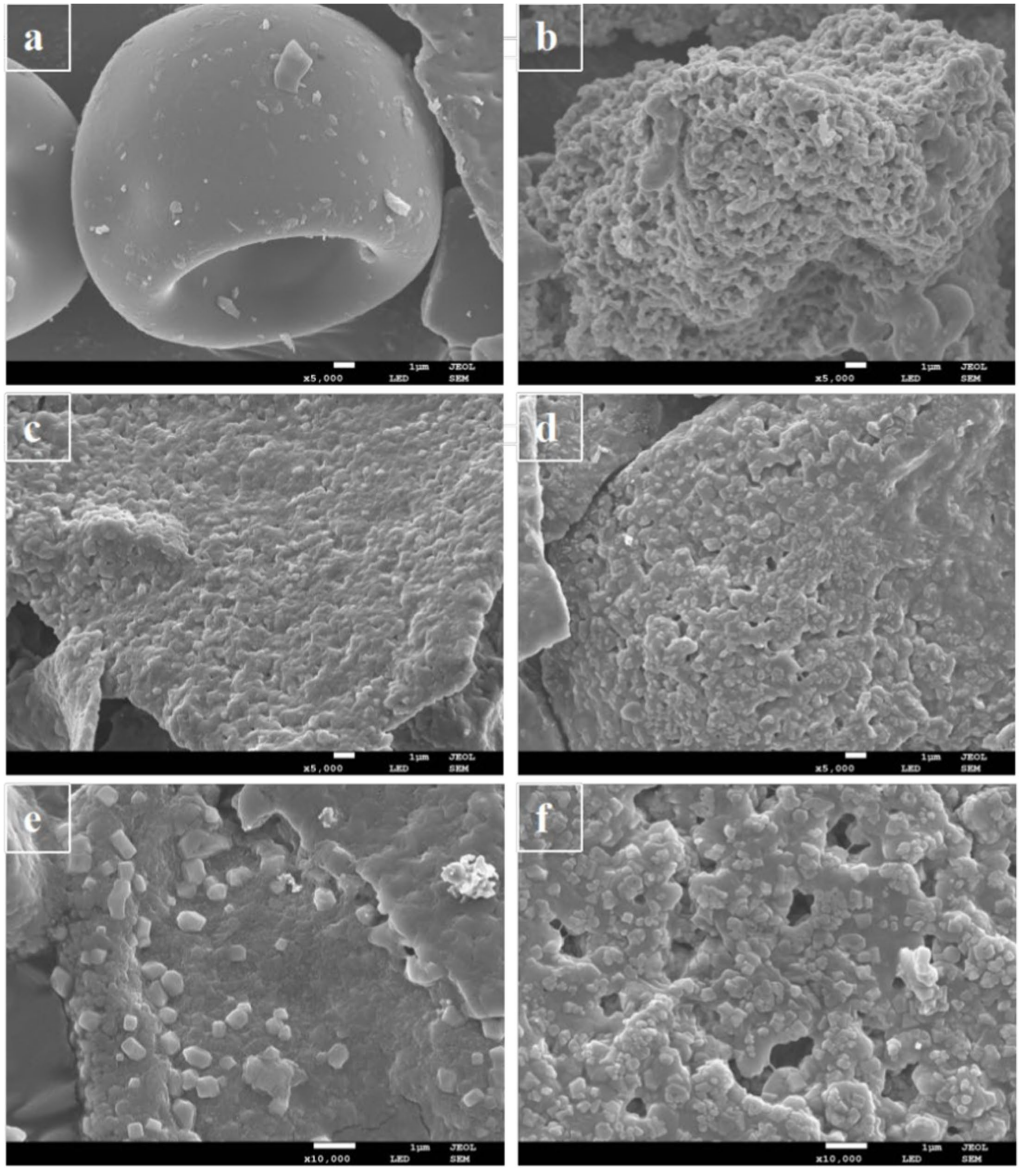
SEM micrographs of **(A)** PC, **(B)** phlorotannin, **(C)** PP (1:1)-5,000x, **(D)** UPP (1:1)-5,000x, **(E)** PP (1:1)-10,000x, and **(F)** UPP (1:1)-10,000x.

SEM analysis is very important for obtaining detailed information on the surface morphology and composition (45). Figure 4 showed SEM images of PC, phlorotannin, PP, and UPP and clearly demonstrated the morphological distinction between PC, phlorotannin, PP, and UPP. PC had large bulks, and PP had many smaller flakes after the addition of phlorotannin, which confirmed the formation of nanosized mixtures in Figure 4C. Phlorotannin cross-linked and aggregated with proteins, indicating that phlorotannin induced PC polymerization, and after ultrasound treatment, the particles were broken into smaller square shapes (Figure 4D), and changes in protein structure may be due to weak interactions. It was evident from Figures 4E,F that the bulk structure of UPP was significantly more than that of PP, potentially as a result of hydrophobic groups being exposed and conformational unfolding may provide more binding sites for phlorotannin. This result might be consistent with the XRD results described above. Liu et al. found that the sample of β-lactoglobulin and chlorogenic acid also changed from a complete sheet structure to fragments by SEM observation after ultrasound (42). In line with the experimental findings, PC and phlorotannin likewise underwent a full sheet structure to fragmentation shift.

### 3.3. *In vitro* digestibility

The effect of polyphenols on protein digestion has been extensively studied, with results varying depending on the interaction. The reaction of PC with phlorotannin, especially covalent cross-linking, was expected to disturb protein digestion. After 60 min of simulated stomach digestion, the samples were added to simulated intestinal digestive solution for intestinal digestion. The electrophoretic patterns of the samples after simulated gastrointestinal digestion of PC and its complex PP, UPP, were shown in Supplementary Figure S1. Ultrasound-assisted phlorotannin modification resisted PC digestion and showed that UPP was more resistant to simulated gastrointestinal digestion. The underlying cause might be the reduced sensitivity of the complex to pepsin, which was observed in a previous study (46).

### 3.4. Free radical scavenging activity

**Figure 5:**
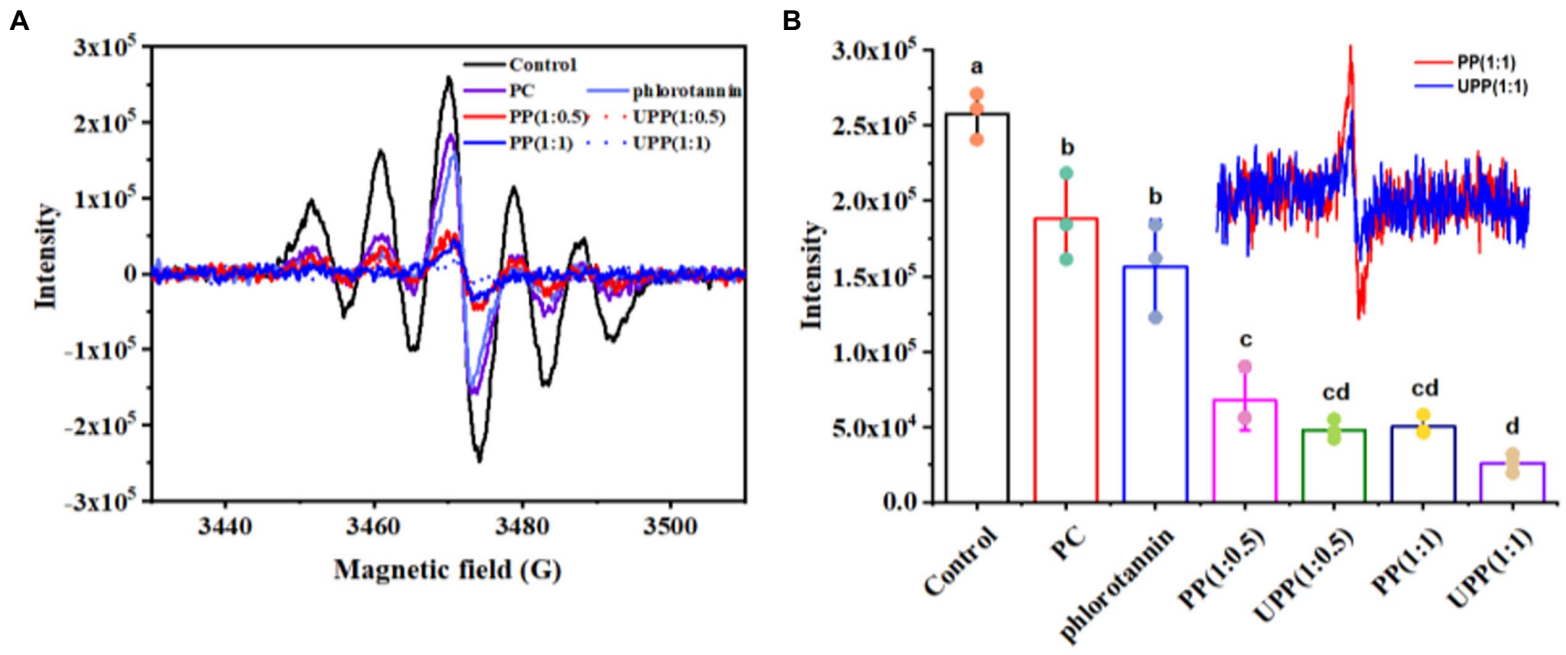
ESR spectra **(A)**, and maximum peak intensities **(B)** of PC, phlorotannin, PP and UPP on DPPH free radical scavenging ability. Different lowercase letters represent significant (*p* < 0.05) differences between groups.

The antioxidant properties of antioxidants are usually measured by the scavenging activity of DPPH and ABTS^+^. The DPPH radical scavenging activity of PC, PP, and UPP was systematically investigated (Figure 5). As shown in Figure 5A, the peak intensities of all samples were lower than those of the control group, indicating that all samples had DPPH radical scavenging activity. As shown in Figure 5B, the peak intensities of UPP after ultrasound treatment were lower than those of untreated complex PP. The activity of PC scavenging DPPH radical was significantly enhanced by ultrasound assistance and the addition of phlorotannin. Some researchers reported that the increased scavenging rate of DPPH free radicals after ultrasound treatment may be related to the cavitation effect during ultrasound treatment, which promoted the unfolding of some proteins and exposed more aromatic amino acids with antioxidant activity (47, 48). Other researchers have reported similar results that the binding of proteins to polyphenols such as catechins, EGCG, and EGC improves the ability of proteins to scour DPPH radicals (49, 50). Supplementary Figure S2 displayed the ABTS^+^ radical scavenging activities of PC, PP and UPP. This trend was demonstrated in Supplementary Figure S2 and appeared similar to the result of DPPH, and showed that the ABTS^+^ radical scavenging activity of UPP after ultrasound treatment was also higher than that of PP. These results suggested that ultrasound treatment might destroy macromolecules, releasing small molecules with higher antioxidant capacity, thereby enhancing free radical scavenging capacity. Liu et al. reported that turbulence and shear generated by ultrasound treatment could break particles and expose more ends which were hydrophobic, thus improving the scavenging activity of mung bean protein hydrolysate against ABTS^+^ radicals (25).

### 3.5. Photoprotection in UVB-induced RMCs oxide damage

**Figure 6:**
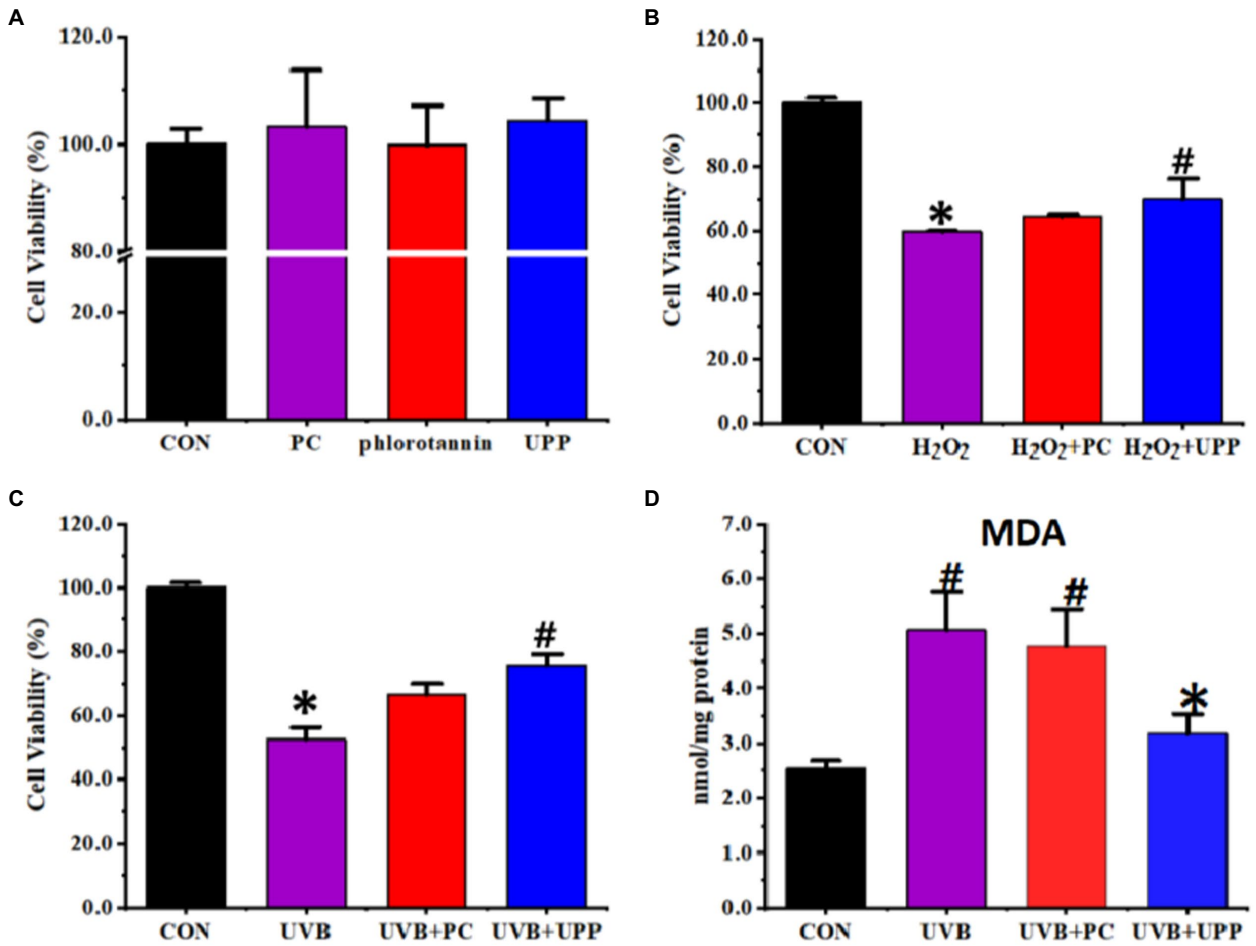
Effects on the RMCs of **(A)** Cytotoxicity, **(B)** cell viability stimulated by hydrogen peroxide, **(C)** cell viability induced by UVB, and **(D)** MDA levels induced by UVB. Different letters represent significant (*p* < 0.05) differences between groups.

The cytotoxicity of PC, phlorotannin, and UPP toward RMCs was evaluated first. As depicted in Figure 6A, the viability of RMCs remained above 90% after incubation for 24 h throughout the studied concentrations. Compared with the control, there were no significant changes in cell viability in the PC, phlorotannin and UPP groups. Therefore, PC, phlorotannin, and UPP did not cause any cytotoxic effects at the studied concentrations. However, as shown in Figures 6B,C, both PC and UPP could improve cell viability in cells stimulated by hydrogen peroxide and induced by UVB.

Next, we evaluated MDA levels. Oxidative stress induced by UVB irradiation has been shown to be converted into components that maintain intracellular lipid homeostasis (51). High-dose light-induced lipid peroxidation products, especially malondialdehyde (MDA), readily attacked macromolecules such as proteins and DNA, making the retina vulnerable to photooxidative damage (52). MDA was considered to be an important parameter reflecting the level of intracellular antioxidant. As shown in Figure 6D, under normal culture conditions, the intracellular MDA content of RMCs was very low, only 2.53 ± 0.14 nmol/mg protein. RMCs underwent lipid peroxidation and produced more lipid peroxide MDA after irradiation with UVB. Without the protection of antioxidants, the MDA content in RMCs rapidly increased to 5.07 ± 0.71 nmol/mg protein. With the intervention of drugs, the intracellular MDA content of RMCs under UVB irradiation decreased (*p* < 0.05).

## 4. Discussion

In this study, PC-phlorotannin was used to prepare UPP by ultrasound treatment. The fluorescence intensity of PC decreased, and the wavelength of PC showed a certain degree of redshift after ultrasound treatment and the binding of phlorotannin. The content analysis of the secondary structure showed that α-helix structures decreased, while β-sheets, β-turns and random coils increased. Studies on the functional properties of PC and associated complexes have highlighted that UPP complexes modified with ultrasound treatment and the binding of phlorotannin have increased denaturation temperature and antioxidant capacity, as well as enhanced resistance to simulated gastrointestinal digestion. The protein-loaded complexes fabricated in this work had good property, herein could be applied to explore protection barriers and vehicles for active functional proteins.

## Data availability statement

The original contributions presented in the study are included in the article/Supplementary material, further inquiries can be directed to the corresponding author.

## Author contributions

HQ: conceptualization, methodology, software, and resources. YB: investigation, writing, and original draft preparation. XL: investigation, visualization, and date curation. YX: visualization and reviewing. YW: visualization, validation, and reviewing. XD: reviewing and editing. All authors contributed to the article and approved the submitted version.

## Funding

This research was funded by the National Key Research and Development Program of China (grant number 2019YFD-0902001), the National Natural Science Foundation of China (no.31972143), Key Science and Technology Program of Liaoning Province (2020JH1/10200001), and Natural Science Foundation of Shandong Province (ZR2021QC168).

## Conflict of interest

The authors declare that the research was conducted in the absence of any commercial or financial relationships that could be construed as a potential conflict of interest.

## Publisher’s note

All claims expressed in this article are solely those of the authors and do not necessarily represent those of their affiliated organizations, or those of the publisher, the editors and the reviewers. Any product that may be evaluated in this article, or claim that may be made by its manufacturer, is not guaranteed or endorsed by the publisher.

## Abbreviations

PC: Phycocyanin
*A. nodosum*: Ascophyllum nodosum
CD: Circular dichroism
XRD: X-ray diffraction
DSC: Differential scanning calorimetry
FTIR spectroscopy: Fourier transform infrared spectroscopy
FBS: Fetal bovine serum
ABTS: 2,2′Azino-bis (3-ethylbenzothiazoline-6-sulfonic acid)
PP: PC-phlorotannin complexes
SDS-PAGE: Sodium dodecyl sulfate-polyacrylamide gel electrophoresis
SEM: Scanning Electron Microscopy
