# Supplementary figures and images for: Ultrasound treatment enhanced the functional properties of phycocyanin with phlorotannin from Ascophyllum nodosum
Source: Front Nutr. 2023 Apr 6;10:1181262. doi: 10.3389/fnut.2023.1181262 (PMC10115965; doi:10.3389/fnut.2023.1181262)

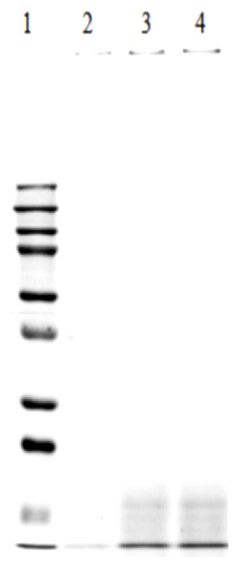

Supplement: Supplementary file 1 [file Image_1.jpg]

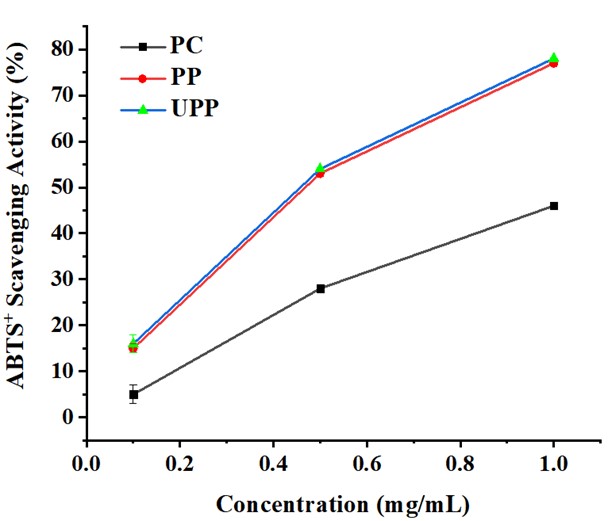

Supplement: Supplementary file 2 [file Image_2.jpg]
